# Analysis of Early EEG Changes After Tocilizumab Treatment in New-Onset Refractory Status Epilepticus

**DOI:** 10.3390/brainsci15060638

**Published:** 2025-06-13

**Authors:** Yong-Won Shin, Sang Bin Hong, Sang Kun Lee

**Affiliations:** 1 Department of Critical Care Medicine, Seoul National University Hospital, Seoul 03080, Republic of Korea; shinwn04@gmail.com; 2 Center for Hospital Medicine, Seoul National University Hospital, Seoul 03080, Republic of Korea; hong.sangbin@gmail.com; 3 Department of Neurology, Seoul National University Hospital and Seoul National University College of Medicine, Seoul 03080, Republic of Korea

**Keywords:** new-onset refractory status epilepticus, tocilizumab, quantitative EEG

## Abstract

**Background/Objectives**: New-onset refractory status epilepticus (NORSE) is a rare neurologic emergency that often requires immunotherapy despite an unclear etiology and poor response to standard treatments. Tocilizumab, an anti-interleukin-6 monoclonal antibody, has shown promise in case reports; however, objective early biomarkers of treatment response remain lacking. We investigated early electroencephalography (EEG) changes following tocilizumab administration in NORSE patients using both quantitative and qualitative analyses. **Methods**: We retrospectively analyzed six NORSE patients who received tocilizumab and underwent continuous EEG monitoring during the period of its administration, following the failure of first- and second-line immunotherapies. Clinical characteristics, treatment history, and EEG recordings were collected. EEG features were analyzed from 2 h before to 1 day after tocilizumab treatment. Quantitative EEG metrics included relative band power, spectral ratios, permutation and spectral entropy, and connectivity metrics (coherence, weighted phase lag index [wPLI]). Temporal EEG trajectories were clustered to identify distinct response patterns. **Results**: Changes in spectral power and band ratios were heterogeneous and not statistically significant. Among entropy metrics, spectral entropy in the theta band showed a significant reduction at 1 day post-treatment. Connectivity metrics, particularly wPLI, demonstrated a consistent decline after treatment. Clustering of subject–channel trajectories revealed distinct patterns including monotonic changes, indicating individual variation in response. Visual EEG review corroborated qualitative improvements in all cases. **Conclusions**: Tocilizumab was associated with measurable early EEG changes in NORSE, supported by visually noticeable EEG changes. Quantitative EEG may serve as a useful early biomarker for treatment response in NORSE and assist in monitoring the critical phase. Further validation in larger cohorts and standardized protocols is warranted to confirm these findings and refine EEG-based biomarkers.

## 1. Introduction

Status epilepticus (SE) is a neurologic emergency characterized by continuous seizures lasting 5 min or more that fail to terminate spontaneously and may result in long-term consequences [1]. Around one-third of SE cases progress to refractory status epilepticus (RSE), which does not respond to multiple antiseizure medications (ASMs) and is associated with high morbidity and mortality [2,3]. Among the refractory cases, new-onset refractory status epilepticus (NORSE) is a rare clinical presentation that occurs in individuals without active epilepsy and clear acute or active structural, toxic, and metabolic causes [4]. Given its devastating and often life-threatening course and the lack of an established set of etiologies, extensive evaluation is recommended for all patients with NORSE [5]. However, a significant proportion of cases remain without a definitive etiology [6,7]. Excluding cryptogenic cases, autoimmune/paraneoplastic and infectious causes are reported as the most common causes.

As inflammation-related etiologies are among the most commonly identified causes, immunotherapeutic agents are considered a potential treatment option for NORSE, even in cryptogenic cases [5]. In autoimmune etiologies, such as anti-leucine-rich glioma-inactivated 1 (LGI1) encephalitis, seizures are often refractory to ASMs alone but respond to immunotherapy [8,9]. Early initiation of immunotherapy is emphasized, as it is associated with favorable outcomes in autoimmune central nervous system (CNS) disorders [9,10,11,12,13]. However, misdiagnosis may expose patients to the potential harms of immunotherapy side effects [14]. Therefore, diagnosis of autoimmune-related encephalopathic conditions requires caution, especially in seronegative (autoantibody-negative) cases [15].

Although NORSE is a poorly defined syndrome, and the mechanisms leading to RSE are not always autoimmune in origin, the role of inflammation in epilepsy and status epilepticus has been supported by previous studies [16,17]. Therefore, immunotherapy remains a potential treatment option for NORSE, which otherwise often results in devastating outcomes. It is known that commonly used first-line immunotherapeutic agents, such as corticosteroids, intravenous immunoglobulin, and plasmapheresis, as well as second-line agents, like rituximab and cyclophosphamide, do not effectively control NORSE with cryptogenic etiology [18]. However, a range of immunotherapeutic options exist that target broader aspects of the immune system beyond antibody-mediated mechanisms [19]. Notably, there are also reports of clinical benefits from immunotherapy in seronegative cases [20,21].

Tocilizumab is a monoclonal antibody targeting the interleukin-6 (IL-6) receptor, initially approved for the treatment of rheumatoid arthritis [22]. Elevated IL-6 levels have been reported in patients with NORSE, and tocilizumab has been associated with clinical improvement in several cases following the failure of first- and second-line immunotherapies [23,24,25,26,27,28].

Electroencephalography (EEG) is the gold standard for assessing seizure activity and is widely used in the SE to evaluate treatment response. In severe conditions, such as NORSE, clinical improvement often requires prolonged observation and is frequently determined by clinical judgment, especially when both clinical signs and visually assessed EEG changes remain ambiguous. Given the frequent vagueness in clinical response and the often cryptogenic nature of NORSE, EEG offers a non-invasive and continuous monitoring tool that may assist in evaluating therapeutic response. In this study, we investigated EEG changes following tocilizumab administration using both quantitative and qualitative measures, aiming to identify early EEG features that may help determine treatment response and guide further management.

## 2. Materials and Methods

### 2.1. Study Subjects

From August 2015 to June 2020, we retrospectively identified patients admitted to the Department of Neurology at Seoul National University Hospital who met the following criteria: (1) new-onset (de novo) status epilepticus refractory to the appropriate dose of two or more ASMs, (2) no identifiable infectious agents or autoantibodies attributable to autoimmune encephalitis during the evaluation, and (3) continuous EEG monitoring initiated prior to and maintained for approximately one day following tocilizumab administration. For patients with NORSE, first-line immunotherapies, including high-dose intravenous methylprednisolone and intravenous immunoglobulin, were administered unless contraindicated. If no adequate clinical improvement was observed, second-line treatment with rituximab was considered. Tocilizumab was initiated in patients with intractable NORSE who failed to respond to these prior immunotherapeutic agents, following the institutional protocol established for treatment-refractory autoimmune encephalitis [29].

### 2.2. EEG Data Acquisition and Preprocessing

EEG data were acquired from 19 scalp electrodes (Fp1, Fp2, Fz, F3, F4, F7, F8, Cz, C3, C4, T7, T8, Pz, P3, P4, P7, P8, O1, and O2) according to the international 10–20 system, using the NicoletOne™ EEG System. From the continuous EEG recordings, eight time points were selected for analysis: 2 and 1 h before and 1, 2, 3, 4, 12 h, and 1 day after tocilizumab administration. Tocilizumab was administered via intravenous infusion over approximately one hour, and its pharmacological effects are known to begin immediately upon completion of infusion. To capture the early neurophysiological response to the IL-6 receptor blockade, we analyzed EEG changes starting from one hour after the end of infusion. Tocilizumab has been reported to reduce fever and clinical improvement within a median of 4 h in cytokine release syndrome following chimeric antigen receptor T cell therapy [30], suggesting a rapid onset of action. Furthermore, blood–brain barrier integrity is often compromised in NORSE [31], which may facilitate the CNS effect of tocilizumab. Based on these considerations, we selected a 1 to 4 h post-infusion window to evaluate immediate EEG changes following tocilizumab administration. Tocilizumab exerts its effects by blocking the IL-6 receptor, thereby inhibiting the downstream Janus kinase/signal transducer and activator of the transcription 3 (JAK/STAT3) signaling pathway and dampening the inflammatory cytokine network. Previous studies have demonstrated suppression of the IL-6-induced phosphorylation of STAT3 and a reduction in membrane-bound IL-6 receptors within 1 day after administration [32]. Accordingly, we also examined EEG changes up to 1 day post-infusion to capture short-term dynamics potentially reflecting early immunological effects of tocilizumab on brain activity. The time point corresponding to the period of tocilizumab administration was excluded to avoid potential misalignment between the recorded and actual administration times. The EEG recorded 1 h prior to tocilizumab administration was designated as the baseline for all comparisons. EEG recordings from 2 h prior to tocilizumab were available for all participants and were included to assist in estimating the range and stability of the baseline.

For each time point, a 5 min segment of artifact-free EEG data was selected based on visual inspection. EEG segments that included the evolution of generalized seizures followed by postictal suppression were excluded. EEG signals were downsampled to 200 Hz to ensure consistency across datasets and then preprocessed using the PREP pipeline [33] implemented in MATLAB R2022a (MathWorks Inc., Natick, MA, USA), which includes notch filtering at 60 Hz to remove line noise, high pass filtering at 1 Hz to eliminate slow drifts, and re-referencing to a robust average reference.

### 2.3. EEG Feature Extraction

EEG data were segmented into consecutive non-overlapping 5 s epochs. Power spectral density (PSD) was computed for each channel and epoch using multitaper methods, covering the following frequency bands: delta (δ, 1–4 Hz), theta (θ, 4–8 Hz), alpha (α, 8–13 Hz), and beta (β, 13–30 Hz). Relative power for each band was calculated by normalizing to the broadband power (1–30 Hz). To further characterize spectral profiles, several power ratios were derived, including the theta-to-alpha ratio (θ/α), theta-to-beta ratio (θ/β), delta-to-alpha ratio (δ/α), slow-to-fast ratio (δ + θ)/(α + β), and delta-to-(alpha + theta) ratio (δ/[α + θ]). EEG signal complexity was assessed using permutation entropy, a noise-robust, time-domain measure previously applied in intensive care unit (ICU) environments to evaluate seizures, anesthetic depth, and levels of consciousness [34,35,36,37]. Spectral entropy was also computed, which is frequency based and more sensitive to band-specific dynamics, with an additional advantage in handling high-frequency noise [38]. EEG feature extraction and all subsequent analysis were performed using MNE-Python (v1.8.0) and AntroPy (v0.1.9) libraries in Python (v3.13.0).

### 2.4. Clustering of Temporal PSD Profiles and Visualization

To characterize the diversity of temporal trajectories of spectral EEG features and signal complexity across channels and subjects, we applied K-means clustering to each EEG channel. For each EEG feature, fold changes relative to the baseline were computed at eight time points (pre-2 h, baseline, 1 h, 2 h, 3 h, 4 h, 12 h, and 1 d) for each subject–channel pair. These fold-change values were arranged into time-series vectors and standardized across the eight time points. K-means clustering was then applied to assign each subject–channel to one of several temporal trajectory clusters. The optimal number of clusters was explored using the silhouette and elbow methods, which yielded variable results depending on the EEG feature. Therefore, we selected *k* = 5 as a representative average, which effectively captured distinct dynamic patterns over time, including consistently increasing or decreasing trends. To further identify clusters exhibiting significant directional trends, we applied the Spearman correlation between the average temporal profile of each cluster and time. A cluster was defined as distinct if its average trajectory showed a monotonic increase or decrease from baseline to 1 day, with a statistically significant Spearman correlation coefficient (*p* < 0.05) in the same direction as the slope. K-means clustering was performed using the scikit-learn library (v1.5.2), and Spearman correlation analysis was conducted using SciPy (v1.14.1).

### 2.5. Functional Connectivity Analysis

To assess functional connectivity, we employed two complementary measures, coherence and the weighted phase lag index (wPLI), which capture both amplitude- and phase-based coupling [39,40]. A multitaper spectral estimator was applied to compute cross-spectral density matrices from non-overlapping 5 s epochs. Connectivity was calculated within five canonical frequency bands: delta (1–4 Hz), theta (4–8 Hz), alpha (8–13 Hz), beta (13–30 Hz), and broadband (1–30 Hz). Connectivity metrics were computed using the “spectral_connectivity_epochs” function from the MNE-Connectivity package (v0.7.0). For each frequency band and method, connectivity values were averaged across all channel pairs to yield a global connectivity measure for each subject at each time point.

### 2.6. Visual Analysis of EEG for Improvement

Raw EEG data were visually assessed to evaluate qualitative improvement considering the following features: (1) reduction in the periodic rhythmic pattern (PRP) in terms of frequency, amplitude, and the presence of plus modifiers or other complex features, (2) epileptiform activity (EA) shifting generalized to more localized patterns, (3) any EA transitioning from continuous to intermittent or transient/disappearing, and/or (4) recovery of background activity from suppression. Visual assessment was conducted for each EEG recording from baseline to all time points at which quantitative EEG analysis was performed. Overall impressions of EEG improvement were independently rated by two board-certified epileptologists (Y.W.S. and S.B.H.), who were blinded to the quantitative EEG results, using a structured 3-point ordinal scale (worsened, no change, improved). In cases of discrepancy, the final classification was determined by consensus. To quantify inter-rater reliability, we calculated the weighted Cohen’s kappa using equal spacing weights, appropriate for ordinal data.

### 2.7. Statistical Analysis

To assess systematic changes in baseline-normalized EEG metrics (Δcoef) over time, we fitted a linear mixed-effects model in which Δcoef as the dependent variable. The time point was included as a categorical fixed effect, and the subject was modeled as a random intercept to account for within-subject correlations. This random effects structure captures inter-individual heterogeneity in baseline EEG patterns and treatment response, allowing each patient to have a unique trajectory. Models were estimated using restricted maximum likelihood (REML) rather than full maximum likelihood, as REML provides more accurate and less biased estimates of variance components in unbalanced or small datasets by separating the estimation of fixed and random effects [41]. The Benjamini–Hochberg correction was additionally applied to model-estimated *p* values to control the false discovery rate in multiple comparisons. As a complementary approach, we also performed non-parametric Friedman tests for repeated measures. The Friedman test assesses whether there are systematic differences in ranked values across multiple time points within subjects, suitable for small-sample longitudinal data. This dual analytical approach was employed to enhance the robustness and reliability of statistical inferences in the context of small, heterogeneous clinical data. When the overall Friedman test was significant, post hoc pairwise comparisons between the baseline and each time point were conducted using the Wilcoxon signed-rank test with the Benjamini–Hochberg correction for multiple comparisons. Statistical significance was determined based on model-estimated *p* values for each time point relative to the baseline. These values were annotated on bar plots to indicate significance levels (* *p* < 0.05, ** *p* < 0.01, *** *p* < 0.001). Analyses were performed in Python using statsmodels (v0.14.4) and visualized with matplotlib and seaborn. Spearman correlation analyses were conducted as described above.

## 3. Results

### 3.1. Clinical Characteristics of the Subjects

A total of six subjects (three men and three women) were included in the analysis. The age of enrolled subjects ranged from 17 to 62 years (Table 1). All but one subject underwent first- and second-line immunotherapies prior to tocilizumab treatment, with commonly used agents including high-dose intravenous methylprednisolone, intravenous immunoglobulin, and rituximab. The time from seizure onset to initiation of first-line immunotherapy ranged from 1 to 8 days, and onset to tocilizumab administration ranged from 6 to 48 days. All subjects showed a favorable clinical response to tocilizumab based on clinical assessment, although the degree of response varied. Long-term outcomes at the last follow-up also differed across subjects, ranging from functional independence to bedridden status.

### 3.2. Change of Quantitative EEG Features After Treatment

**Figure 1:**
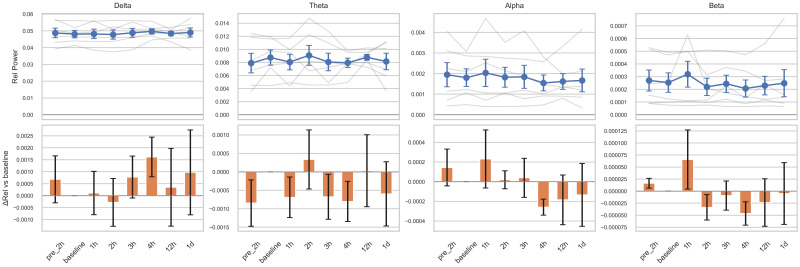
Relative EEG power changes across frequency bands before and after tocilizumab treatment. For each frequency band, the top row illustrates individual subject trajectories (semi-transparent gray lines) and the group mean ± standard error of the mean (SEM), shown as blue circles with error bars. Time points on the *x*-axis are pre-2 h (2 h before tocilizumab), baseline (1 h before tocilizumab), 1 h, 2 h, 3 h, 4 h, 12 h, and 1 d (1 day after tocilizumab). The bottom row displays bar plots of the change in relative power (ΔRel), calculated as the mean difference from each subject’s baseline value and shown with orange bars ± SEM. The baseline thus serves as the statistical reference point for all comparisons. Statistical comparisons were performed using linear mixed-effects models with subject-level random intercepts. No statistically significant changes from the baseline were observed in any time point or frequency band.

**Figure 2:**
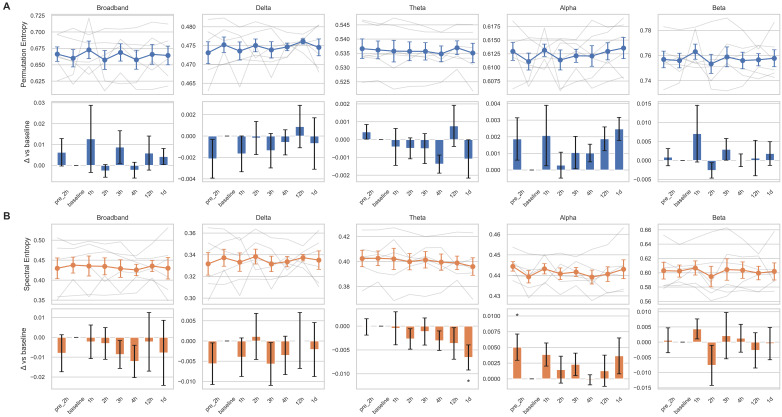
Temporal changes of entropy measures across frequency bands before and after tocilizumab treatment. Permutation entropy (**A**) and spectral entropy (**B**) are shown for five canonical frequency bands: broadband, delta, theta, alpha, and beta. For each entropy measure, the top row displays temporal trajectories across eight time points—pre-2 h (2 h before tocilizumab), baseline (1 h before), 1 h, 2 h, 3 h, 4 h, 12 h, and 1 d (1 day after)—with individual subject traces in semi-transparent gray lines and the group mean ± standard error of the mean (SEM) overlaid in colored markers (blue for **A**, orange for **B**). The bottom row shows the change in entropy relative to the baseline (Δ vs. baseline), represented as bar plots of the mean difference with SEM. Bars are color coded by entropy type (blue for permutation entropy, orange for spectral entropy), and statistical significance was assessed using linear mixed-effects models with subject-level random intercepts. Asterisks indicate comparisons that reached nominal significance (* *p* < 0.05).

Changes in relative power across the four frequency bands following tocilizumab treatment varied among subjects (Figure 1). Although delta band power increased at four time points from 3 h onward, and other bands showed decreasing trends between 2 and 4 h, these changes were not statistically significant. These patterns may reflect transitions from active epileptiform activity to treatment-induced suppression or diffuse slowing. However, substantial heterogeneity in ongoing seizure patterns, disease severity, and individual variability in treatment response may have contributed to the lack of statistical significance. The five power ratio metrics (θ/α, θ/β, δ/α, slow/fast, and δ/[α + θ]) also demonstrated nonsignificant results with inter-individual variability (Figure S1). Complexity analysis using entropy measures revealed a trend toward decreasing spectral entropy in the theta band, with a significant reduction observed at 1 day post-treatment compared to the baseline (*p* = 0.023; Figure 2). However, this result did not remain significant after correction for multiple comparisons (adjusted *p* = 0.16). The Friedman test did not reveal a significant overall change across time points in any of the measures.

### 3.3. Temporal Trajectories of Clusters of Subject–Channel Pairs After Treatment

**Figure 3:**
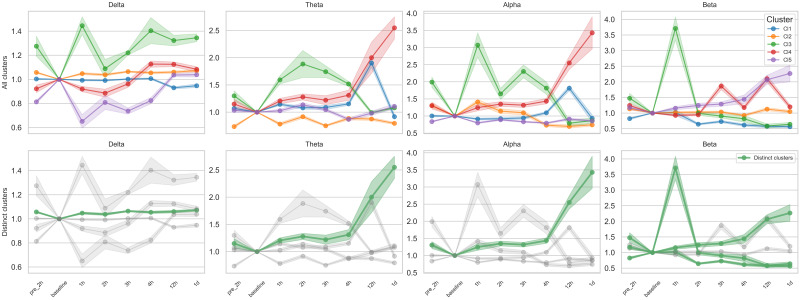
K-means clustering of temporal trajectories in relative band powers. Each panel shows the fold change in relative power, calculated as the ratio of each time point value to the baseline, across eight time points (pre-2 h to 1 day after tocilizumab). K-means clustering (k = 5) was applied to subject–channel-specific trajectories for each frequency band (delta, theta, alpha, beta); details of the clustering procedure are provided in the Methods section. In the top row, all identified clusters (i.e., five per frequency band) are plotted separately, with color-coded lines representing cluster-level means and shaded areas indicating ± standard error of the mean (SEM). In the bottom row, only distinct clusters, defined as those exhibiting monotonic increases or decreases over time with statistically significant Spearman correlation (*p* < 0.05), are highlighted in green, while non-distinct clusters are shown in gray for reference. A total of six distinct monotonic trends were identified across bands: one each in delta, theta, and alpha and three in beta.

Given the considerable variability in EEG feature changes, we performed clustering on all subject–channel EEG data to identify temporal patterns with consistent trajectories. K-means clustering of 114 subject–channel combinations (6 subjects × 19 channels) revealed several distinct patterns, including overall increasing or decreasing trends over time, transient deviation followed by recovery, and non-directional or variable patterns (Figure 3, Figures S2 and S3). Among these, we identified distinct clusters characterized by monotonic increases or decreases in EEG features across time points. These clusters demonstrated significant Spearman correlations between mean feature values and time for multiple EEG metrics, including relative band powers, band ratios, and entropy measures.

### 3.4. Connectivity Changes over Time After Tocilizumab Treatment

**Figure 4:**
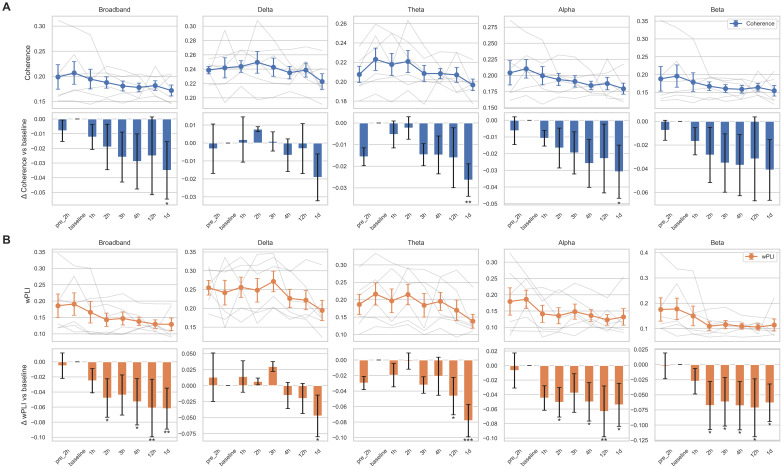
Temporal changes of connectivity metrics across frequency bands. Coherence (**A**) and the weighted phase lag index (wPLI; (**B**)) are shown for five canonical frequency bands: broadband, delta, theta, alpha, and beta. For each connectivity measure, the top row displays temporal trajectories across eight time points (pre-2 h to 1 day after tocilizumab), with individual subject traces in semi-transparent gray lines and the group mean ± standard error of the mean (SEM) overlaid in colored markers (blue for (**A**), orange for (**B**)). The bottom row shows the change in connectivity relative to the baseline (Δ vs. baseline), represented as bar plots of the mean difference with SEM. Bars are color coded by connectivity type (blue for coherence, orange for wPLI), and statistical significance was assessed using linear mixed-effects models with subject-level random intercepts. Asterisks indicate comparisons that reached nominal significance: * *p* < 0.05, ** *p* < 0.01, *** *p* < 0.001.

We additionally analyzed longitudinal changes in functional connectivity using EEG coherence and wPLI. Interestingly, in contrast to other quantitative EEG features, both coherence and wPLI exhibited a significant decreasing trend over time, with more pronounced changes observed in wPLI (Figure 4). While individual variability in EEG connectivity changes remained, most subjects showed patterns generally consistent with the overall downward trend. After correction for multiple comparisons, coherence at 1 day post-tocilizumab in the theta band remained significant (*p* = 0.012). For wPLI, changes remained significant or marginally significant at 4 h (*p* = 0.0502), 12 h (*p* = 0.027), and 1 day (*p* = 0.027) in the broadband range and at 1 day in the theta band (*p* = 0.002) compared to the baseline. The Friedman test revealed a significant effect of time for coherence in the theta band (*p* = 0.0065) and for wPLI in both the delta (*p* = 0.0251) and theta bands (*p* = 0.0117). However, post hoc comparisons failed to identify any specific time point with a significant difference from the baseline, suggesting that the observed effects may be diffuse, modest, or driven by cumulative trends rather than discrete shifts.

### 3.5. Qualitative EEG Changes and Correlation with Quantitative Measures

Visual assessment of raw EEG revealed at least subtle improvements in all subjects, with changes observed as early as 1 to 4 h following tocilizumab administration (Table 2). These improvements included a reduction in PRPs and other EAs, as well as a decreased frequency of PRPs evolving into generalized seizures. The weighted Cohen’s kappa for inter-rater agreement on visual EEG response ratings was 0.74, indicating substantial agreement. In two subjects, EEG showed re-worsening; however, overall improvement was maintained in all subjects at least until the 12 h time point. Although these improvements were not sufficiently robust to be considered clinically transformative, the observed changes suggest that tocilizumab may stabilize or mitigate the progression of disease. These findings are consistent with previous reports supporting its potential efficacy in NORSE [23,28].

## 4. Discussion

In this study, we investigated early EEG response to tocilizumab in patients with NORSE, using both quantitative methods and qualitative analysis. The results suggest that tocilizumab induces measurable neurophysiological changes within hours of administration, including spectral power, entropy, and functional connectivity metrics. Although spectral power and entropy metrics initially did not show significant changes, subject–channel-based grouping of EEG showed several distinct groups showing monotonic linear or wax and waning patterns. These changes are observed along with visual EEG improvement, supportive of potentially early neurophysiologic markers of treatment response.

The observed EEG effects may be interpreted in the context of IL-6’s established role in neuroinflammation and epileptogenesis. Previous studies have demonstrated the mechanistic link between proinflammatory cytokines and the alteration of EEG activity in various neurologic conditions. Epilepsy, and status epilepticus, is one of the representative neurologic conditions strongly associated with neuroinflammation that proinflammatory cytokines such as IL-6, tumor necrosis factor-alpha (TNF-α), and IL-1β have been shown to be upregulated in microglia, astrocytes, and neurons of epileptic tissue specimens [16,42,43]. Elevated IL-6 levels have been reported to be consistently observed in patients with NORSE [23,26,27,44]. Preclinical models have shown that IL-6 enhances glutamatergic transmission and reduces GABAergic inhibition, facilitating excitatory neurotransmission and sustained seizures [45,46]. The IL-6 receptor blockade with tocilizumab has shown benefit in mitigating neuroinflammation in NORSE and related conditions [23,28,29].

Quantitative EEG measures are increasingly recognized as valuable tools for detecting seizures and monitoring dynamic changes in brain function in critically ill patients [47,48]. The most widely used measure is spectral power density. Critically ill patients often show periodic discharges (PDs), which could be a part of status epilepticus or represent an ictal–interictal continuum. Chen et al. conducted spectral power analysis of PDs in 72 patients and found that higher-power PDs were much more likely to be associated with seizures, whereas patients with very low-power PDs often had no further seizures [49]. However, conventional spectral power analyses can be insufficient for capturing the full complexity of EEG dynamics and often necessitate additional metrics to offer deeper insights into cerebral pathophysiology.

Among these advanced metrics, functional connectivity measures, such as coherence and wPLI, can reveal pathological network synchrony associated with seizure propagation. A decrease in these connectivity indices may, therefore, reflect a disruption of such aberrant networks in response to treatment. Brazier in 1972 used coherence to track seizure spreading across brain regions [50]. Song et al. also characterized a coherence pattern during the pre-spike, spike, and post-spike intervals in five patients [51]. Mao et al. used the phase lag index to show a significant increase in frontotemporal connectivity in the theta band in temporal lobe epilepsy, which was correlated with seizure severity [52]. Ergot et al. found that patients with focal epilepsy who underwent epilepsy surgery had a higher likelihood of achieving seizure freedom when there was increased regional connectivity within the resection site [53]. Hwang et al. analyzed interictal EEGs from patients with epilepsy treated with monotherapy and reported that functional connectivity features were predictive of medication refractoriness, with the gamma frequency band demonstrating the highest performance [54]. In this context, a reduction in connectivity measures in NORSE may indicate the attenuation of pathological hypersynchrony in a highly epileptogenic state. In our data, reductions in connectivity, especially wPLI, were observed consistently in the broadband, theta, and delta bands. As these changes coincided temporally with the expected onset of action of tocilizumab, they suggest early neurophysiological responses to immunotherapy.

Among all metrics evaluated, theta band and broadband wPLI demonstrated the most robust and statistically significant change following treatment, even after correction for multiple comparisons. In contrast, entropy-based features showed weaker and more variable trends and did not survive correction for multiple comparisons or the Friedman test. Therefore, wPLI may serve as the most promising single-feature EEG biomarker for capturing early neurophysiological responses to anti-inflammatory treatment in NORSE. However, due to individual variability and our small sample size, it remains difficult to define a fixed cutoff value for wPLI that would enable clinical interpretation beyond general trends. Future studies with larger datasets are necessary to derive data-driven wPLI thresholds linked to clinical outcomes. Ultimately, prospective validation is needed to confirm the reproducibility of this metric across subjects, EEG acquisition conditions, and clinical settings and to determine whether this biomarker can be generalized to other forms of SE or autoimmune encephalitis. Given the feasibility of computing wPLI from routine EEG recordings, this feature is well suited for integration into prospective clinical trial protocols as a promising early neurophysiological biomarker of treatment response.

Entropy and complexity-based measures provide a complementary perspective by quantifying the predictability or irregularity of brain activity. Seizure states, characterized by excessive neuronal synchrony, often lead to more regular EEG patterns and hence reduced entropy. A previous report showing a decrease in EEG entropy in seizures is in line with this concept [55]. However, another study using distribution entropy (DistEn) found that ictal EEG segments actually had higher entropy than both normal and interictal segments [56]. These findings imply that a seizure involves a complex reconfiguration of brain dynamics and that certain aspects become more ordered while others may become more chaotic. The machine learning approach demonstrated that entropy can be utilized to detect seizures that capture these complex features [57,58].

In our cohort, the absence of significant global trends in many quantitative EEG features may not solely indicate a lack of treatment effect but rather reflect a spectrum of individualized responses to immunotherapy. Through subject–channelwise clustering of EEG data, we identified diverse temporal patterns of change following tocilizumab administration. Some clusters showed monotonic increases or decreases, while others exhibited transient fluctuations or delayed responses. These patterns were not reducible to noise but represented physiologically plausible heterogeneity across frequency bands, anatomical locations, and patient trajectories. Previous studies have reported significant changes in specific EEG features, spectral bands, or channels, which also applies to the cited studies [47,48,50]. Such findings may, in part, be attributable to inter-individual variability, where divergent individual responses obscure clear group-level patterns. The etiology of NORSE is heterogeneous, and corresponding MRI findings also reveal marked inter-individual differences and temporal evolution [59]. Considering the known biological variation in quantitative EEG measures across different brain lesions and disease conditions [60,61,62], this variability highlights that interpreting quantitative EEG through the lens of uniform increase or decrease is overly simplistic in complex clinical conditions, such as NORSE. Rather, our findings advocate for tailored classification of EEG trajectories and context-sensitive interpretation.

Clustering-based classification of EEG trajectories may assist in real-time decision-making by stratifying patients according to their neurophysiologic response profiles. For example, when some, but not necessarily all, features such as spectral power, entropy, or connectivity show monotonic changes within specific cluster patterns, a weighted summation of these trends, taking into account their direction and strength as well as the heterogeneity in involved regions, disease severity, and etiology, may help estimate the likelihood of response to immunotherapy. Conversely, patients with weak or inconsistent trends might prompt clinicians to escalate therapy or consider alternative interventions. These trajectory clusters could be incorporated into neurophysiological monitoring protocols in the ICU to provide an additional marker of the patient’s brain state, supplement EEG interpretation, and aid early prognostication in NORSE.

In our study, we selected *k* = 5 for trajectory clustering based on empirical evaluation of multiple *k* values. While meaningful monotonic patterns were consistently observed across commonly used values (*k* = 3, 4, and 5), we selected *k* = 5, as it provided the best balance between granularity and interpretability without overcomplicating the visual presentation. Although the choice of *k* remains a heuristic decision in this preliminary setting, we believe that the patterns observed under *k* = 5 are physiologically meaningful and clinically informative. Further studies with larger patient cohorts and external validation will be needed to improve the clustering approach and better determine the most appropriate number of clusters. In addition, future work may consider incorporating biological interpretability and clinical relevance when selecting the clustering parameters. These efforts could help improve the consistency and practical usefulness of quantitative EEG analysis in NORSE and similar conditions.

Our findings suggest that quantitative EEG measures can serve as an early, objective biomarker of treatment response in patients with NORSE. In critical care settings, especially in NORSE, clinical assessments are confounded by sedation, mechanical ventilation, and profound neurological impairment, which limit conventional clinical scales to detect subtle improvements. Widely used tools, such as the Coma Recovery Scale—Revised (CRS-R) and the Glasgow Coma Scale (GCS), are not applicable or sensitive in deeply comatose patients undergoing ongoing status epilepticus. Traditional indicators of response, including overt arousal or seizure cessation, frequently lag behind underlying neurophysiological recovery, rendering them insufficient for early treatment evaluation. While cerebrospinal fluid analysis offers some insights, it is invasive and not suited for repeated evaluation. EEG is a non-invasive and continuously available modality that directly reflects cortical activity in real time. However, the interpretation of subtle EEG changes through visual inspection alone is inherently subjective. Quantitative EEG measures such as spectral power, entropy, and functional connectivity offer a more sensitive and reproducible means to detect early neurophysiological changes in response to immunotherapy, particularly in critically ill patients where overt clinical improvement may be delayed or obscured. Building on this, our findings suggest that the clustering of temporal EEG feature trajectories and connectivity changes, such as wPLI, may help guide early treatment evaluation and support real-time therapeutic decision-making in ICU settings, including escalation or the adjustment of immunotherapy when conventional assessments are unreliable.

Clinical trials assessing immunotherapeutic agents, such as tocilizumab, in NORSE are logistically and ethically challenging given the acuity, rarity, and severity of the disease. Moreover, recent observational studies support the use of immunotherapy in NORSE and seronegative encephalitis [21,28,63]; clinical outcomes are often evaluated at delayed intervals and after exposure to multiple agents, making it difficult to isolate the effects of a specific treatment, such as tocilizumab. In this context, EEG-based biomarkers may serve as valuable surrogate endpoints for detecting treatment response in future studies where conventional clinical measures are delayed, nonspecific, or confounded by concurrent interventions. Despite considerable inter-individual variability, our analysis quantified and clustered temporal EEG trajectories, revealing distinct patterns of response to tocilizumab. Such data-driven classification may facilitate the development of EEG-derived biomarkers that serve as early indicators of treatment effect and surrogate endpoints in future clinical trials.

This study has several limitations. First, the sample size was very small (*n* = 6), which limits the statistical power to detect subtle effects and reduces the generalizability of the findings. Given the rarity of NORSE, particularly cases treated with tocilizumab, our cohort size reflects the real-world clinical constraint. However, the limited number of cases, combined with heterogeneity in clinical presentation and outcomes, may have introduced variability that obscures consistent EEG patterns or treatment responses. Second, the study lacked a control group, and, therefore, the observed EEG changes cannot be conclusively attributed to IL-6 inhibition independent of the potential effects of concurrent treatments, such as antiseizure medications and other immunotherapies. We attempted to mitigate this by evaluating EEG changes serially within a short time window surrounding tocilizumab administration. Due to the severity and rarity of NORSE, enrolling an appropriate control group in the acute setting is ethically and practically challenging. Nonetheless, future prospective studies with larger cohorts and the inclusion of control groups or comparator arms are necessary to improve statistical robustness and better isolate treatment effects. Finally, long-term functional outcomes were variable, suggesting that early EEG responses may not consistently correlate with clinical recovery. Further research is needed to identify EEG biomarkers that can reliably predict which patients are most likely to benefit from IL-6-targeted therapies.

## 5. Conclusions

This study provides preliminary evidence that tocilizumab induces early and measurable changes in EEG features in patients with NORSE, including reductions in functional connectivity and other quantitative EEG alterations suggestive of the modulation of hyperexcitable neural networks. Qualitative EEG improvements further support its potential to stabilize disease progression, although robust clinical benefits remain difficult to establish. These findings highlight the potential utility of quantitative EEG as a complementary tool for evaluating response to immunotherapy. Further investigations involving larger cohorts and standardized protocols are warranted to validate these findings and to explore the prognostic value of early EEG changes in guiding immunotherapeutic strategies for this devastating condition.

## Figures and Tables

**Table 1:** Demographic and clinical characteristics of the study population.

	Subject 1	Subject 2	Subject 3	Subject 4	Subject 5	Subject 6
Age of onset (years)	24	61	62	47	36	17
Sex	F	F	M	M	M	F
Etiology	Cryptogenic	Cryptogenic	Cryptogenic	Cryptogenic	Cryptogenic	Cryptogenic
Recent history of infections/vaccination	Influenza infection	(-)	Influenza vaccination	(-)	(-)	(-)
Prodromal symptom	Fever	Fever	Fever, myalgia	Behavioral change, fever	Headache, chills	Fever
Pre-existing neurological/autoimmune disorders	(-)	(-)	(-)	(-)	(-)	(-)
Other comorbidities	(-)	(-)	(-)	Chronic hepatitis B, chronic alcoholism	(-)	(-)
CSF profiles						
CSF protein level (mg/dL)	52	58	124	13	258	83.3
CSF leukocyte level (cells/μL)	1	4	2	63	272	9
Brain MRI findings	T2 HSI at the bilateral cerebral hemisphere	T2 HSI at the bilateral hippocampi and left frontal lobe	T2 HSI at the bilateral hippocampi	Not remarkable	Diffuse leptomeningeal enhancement	Not remarkable
Treatment						
Time from SE onset to immunotherapy (days)	2	3	1	2	8	2
Time from SE onset to tocilizumab administration (days)	48	35	13	6	16	17
Dose of tocilizumab	4 mg/kg	4 mg/kg	4 mg/kg	4 mg/kg	6 mg/kg	4 mg/kg
Immunotherapeutic agents used before tocilizumab treatment	mPd, IVIg, RTX	mPd, IVIg, RTX	mPd, IVIg, RTX	mPd, IVIg, RTX	mPd, IVIg	mPd, IVIg, RTX
Concurrent ASMs on tocilizumab treatment	fPHT, LCM, LEV, PB, TPM, VPA	fPHT, LCM, LEV, PB, PGB, TPM	fPHT, LCM, OXC, PER, PGB, VPA	fPHT, LEV, PB, PER, TPM	fPHT, LEV, PB, PER, PGB, TPM	CLB, LEV, PB, PER, PGB, TPM
Outcome measures						
Length of intensive care unit stay (days)	72	18	5	12	18	8
Length of hospital stay (days)	442	143	50	78	118	128
Clinical outcome at discharge	5	5	5	3	4	5
mRS at 6 months	5	5	4	3	4	5
mRS at 1 year or last follow-up	5	5	4 (10 months)	2	1	5 (8 months)

(-), none evident; CSF, cerebrospinal fluid; MRI, magnetic resonance imaging; HSI, high signal intensity; ASM, antiseizure medication; fPHT, phenytoin; LCM, lacosamide; LEV, levetiracetam; PB, phenobarbital; TPM, topiramate; VPA, valproic acid; PGB, pregabalin, OXC, oxcarbazepine; PER, perampanel; mPd, methylprednisolone; IVIg, intravenous immonoglobulin; RTX, rituximab; mRS, modified Rankin scale.

**Table 2:** Qualitative EEG changes over time after tocilizumab treatment.

	Subject 1	Subject 2	Subject 3	Subject 4	Subject 5	Subject 6
Baseline	-	-	-	-	-	-
1 h	S (S/S)	I (I/I)	S (S/S)	S (S/S)	S (S/S)	I (I/I)
2 h	I (I/I)	I (I/I)	S (S/S)	I (I/I)	S (S/S)	W (W/W)
3 h	S (I/S)	S (S/I)	S (I/S)	I (I/I)	S (S/S)	I (I/I)
4 h	I (I/S)	S (S/S)	I (I/S)	S (S/W)	S (S/S)	S (S/W)
12 h	I (I/S)	I (I/I)	I (I/I)	I (I/I)	S (S/S)	S (S/S)
1 d	I (I/I)	S (S/S)	W (W/W)	W (W/W)	S (S/S)	I (I/I)

Values are shown as consensus (Rater 1/Rater 2). For example, I (W/I) indicates a final consensus of I, with initial ratings of W and I, respectively. EEG, electroencephalography; S, not much different from previous EEG; I, slightly improved; W, slightly worsened.

## Data Availability

The raw data supporting the conclusions of this article will be made available by the authors upon request.

## Supplementary Materials

The following supporting information can be downloaded at https://www.mdpi.com/article/10.3390/brainsci15060638/s1, Figure S1: Temporal changes of spectral band ratio metrics; Figure S2: K-means clustering of temporal trajectories in spectral band ratio metrics; Figure S3: K-means clustering of temporal trajectories in permutation entropy; Figure S4: K-means clustering of temporal trajectories in spectral entropy.

## Abbreviations

The following abbreviations are used in this manuscript:

SE	Status Epilepticus
RSE	Refractory Status Epilepticus
ASM	Antiseizure Medication
NORSE	New-Onset Refractory Status Epilepticus
LGI1	Leucine-Rich Glioma-Inactivated 1
CNS	Central Nervous System
IL-6	Interleukin-6
EEG	Electroencephalography
PSD	Power Spectral Density
ICU	Intensive Care Unit
wPLI	Weighted Phase Lag Index
PRP	Periodic Rhythmic Pattern
EA	Epileptiform Activity
PD	Periodic Discharge
DistEn	Distribution Entropy
TNF-α	Tumor Necrosis Factor-Alpha
IL-1β	Interleukin-1 Beta
CRS-R	Coma Recovery Scale—Revised
GCS	Glasgow Coma Scale
CSF	Cerebrospinal Fluid
MRI	Magnetic Resonance Imaging
HSI	High Signal Intensity
fPHT	Fosphenytoin
LCM	Lacosamide
LEV	Levetiracetam
PB	Phenobarbital
TPM	Topiramate
VPA	Valproic Acid
PGB	Pregabalin
OXC	Oxcarbazepine
PER	Perampanel
mPd	Methylprednisolone
IVIg	Intravenous Immunoglobulin
RTX	Rituximab
mRS	Modified Rankin Scale
SEM	Standard Error of the Mean
