# Supplementary material for: Analysis of Early EEG Changes After Tocilizumab Treatment in New-Onset Refractory Status Epilepticus
Source: Brain Sci. 2025 Jun 13;15(6):638. doi: 10.3390/brainsci15060638 (PMC12190564; doi:10.3390/brainsci15060638)
Supplement: Supplementary file 1 [file brainsci-15-00638-s001.zip › brainsci-3667480-supplementary.pdf]

# Supplementary Figures

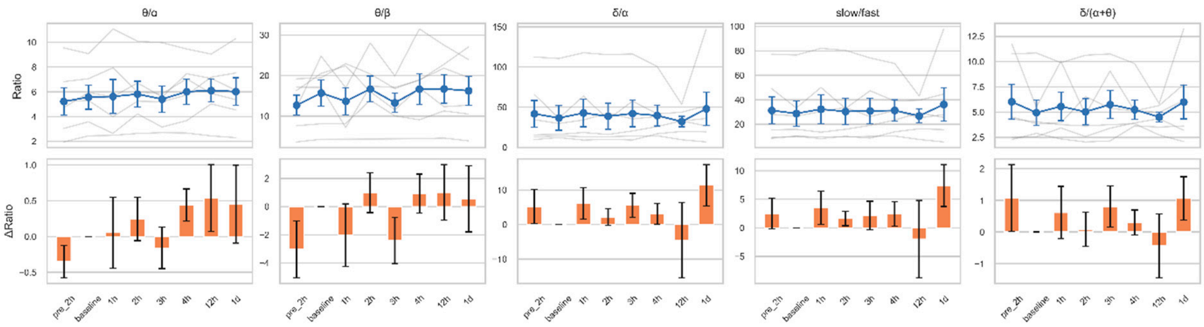


**Figure S1. Temporal changes of spectral band ratio metrics.**

Five spectral band ratio metrics are demonstrated for eight time-points, pre-2 h (2 hours before tocilizumab), baseline (1 hour before tocilizumab), 1 h, 2 h, 3 h, 4 h, 12 h, and 1 d (1 day after tocilizumab), with mean ± SEM as blue circles with error bars and individual subject traces as semi-transparent gray lines. Bar plots in the bottom row show changes in each ratios from baseline (ΔRatio), with error bars denoting SEM.

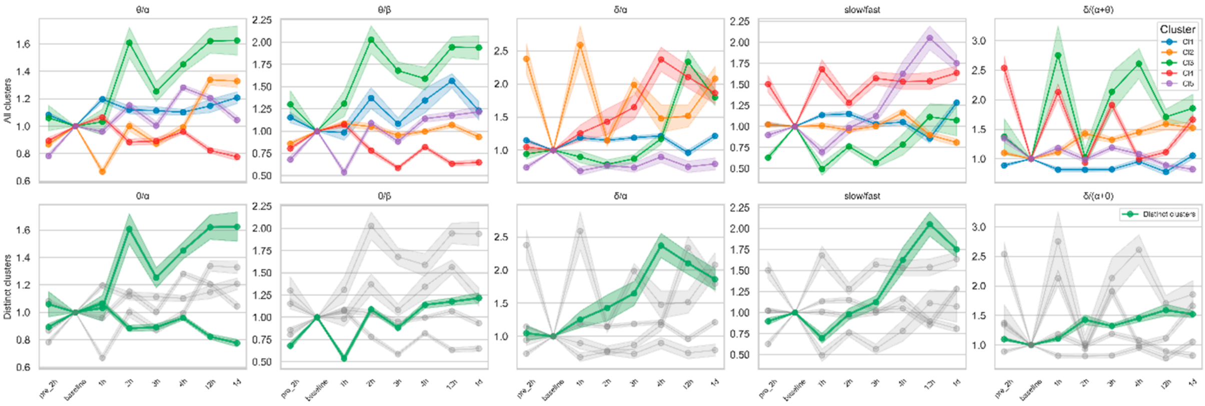


**Figure S2. K-means clustering of temporal trajectories in spectral band ratio metrics.**

Each panel displays the fold-change relative to baseline of spectral band ratio metrics across eight time points (pre-2 h to 1 day), clustered using K-means based on subject–channel-specific trajectories. Top panels show all cluster trajectories; bottom panels highlight distinct clusters characterized by specific temporal patterns (e.g., sustained increase or decrease), as defined in the Methods section. Lines represent cluster means; shaded areas indicate ± SEM.

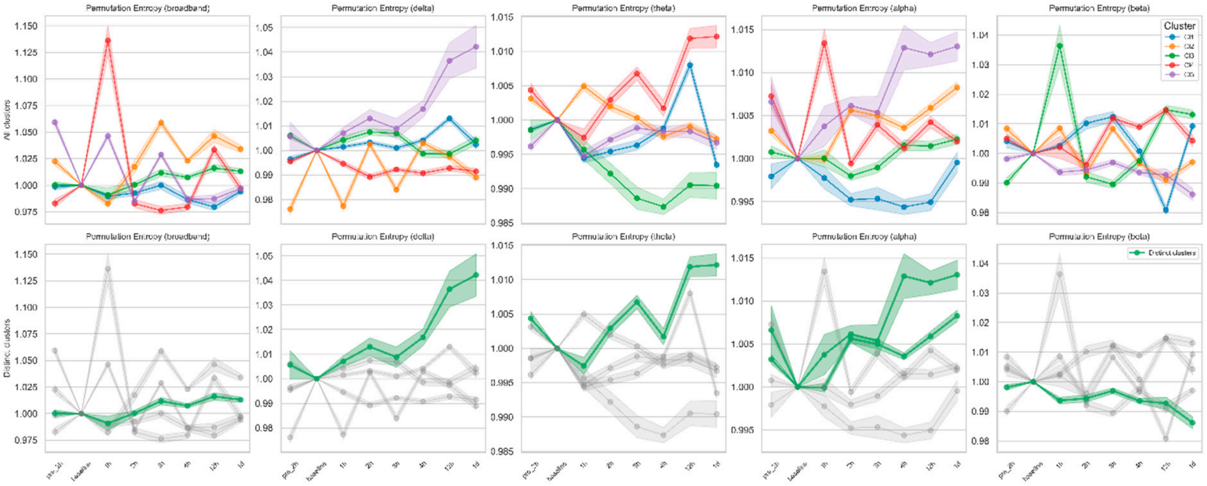


**Figure S3. K-means clustering of temporal trajectories in permutation entropy.**

Each panel shows the fold-change relative to baseline in permutation entropy across eight time points, clustered using K-means based on subject–channel-specific trajectories. Top panels display all cluster trajectories; bottom panels highlight clusters with notable temporal patterns, such as monotonic or biphasic changes. Lines indicate cluster means; shaded regions represent ± SEM.

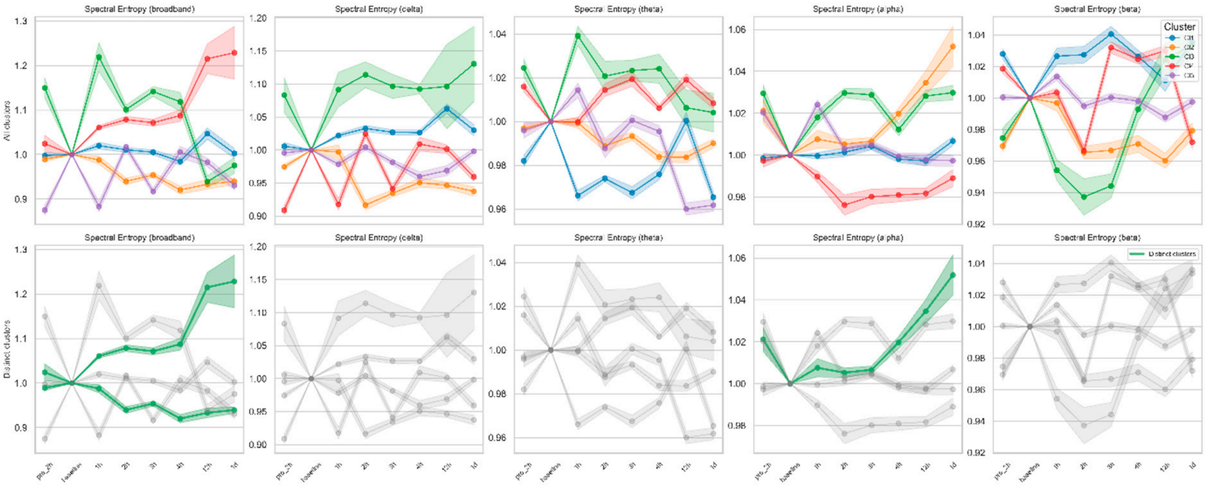


**Figure S4. K-means clustering of temporal trajectories in spectral entropy.**

Each panel shows the fold-change relative to baseline in spectral entropy across eight time points, clustered using K-means based on subject–channel-specific trajectories. Top panels display all cluster trajectories; bottom panels highlight clusters with notable temporal patterns, such as monotonic or biphasic changes. Lines indicate cluster means; shaded regions represent ± SEM.
